# Knowledge, attitudes and practices of nurses regarding maternal nutrition in pregnant women at a large hospital and filter clinics, Lesotho

**DOI:** 10.4102/hsag.v27i0.1768

**Published:** 2022-02-14

**Authors:** Tsiame M. Mekhoa, Nomaxabiso M. Mooi, Olivia B. Baloyi

**Affiliations:** 1 Department of Nursing, Faculty of Health Sciences, Walter Sisulu University, Mthatha, South Africa; 2 Department of Nursing, Faculty of Health Sciences, University of KwaZulu-Natal, Durban, South Africa

**Keywords:** attitude, filter clinics, knowledge, large hospital, maternal nutrition, nurses, practices

## Abstract

**Background:**

Adequate nutritional knowledge, positive attitudes, and good practices of nurses regarding maternal nutrition of women during pregnancy are fundamental for maternal and foetal well-being.

**Aim:**

This study aimed to determine the knowledge, attitudes, and practices of nurses regarding maternal nutrition in pregnant women.

**Setting:**

A large hospital and its filter clinics in Lesotho.

**Methods:**

A descriptive cross-sectional research design was used for this study. The data were collected from 120 nurses working in the reproductive health department of a large hospital and filter clinics, using a self-administered questionnaire. The research was conducted in accordance with the Declaration of Helsinki.

**Results:**

The nurses showed good knowledge and practices, and positive attitudes regarding maternal nutrition, 88.3%, 99.2% and 62.5%, respectively. There was a significant relationship between attitude and practice, with a correlation coefficient of *r* = 296, *n* = 120, and *p* = 0.001.

**Conclusion:**

The results of this study show that nurses’ practices were associated with their attitudes regarding maternal nutrition, meaning their attitudes regarding maternal knowledge transcends their related knowledge.

**Contribution:**

The study contributes to the body of literature about the knowledge, attitudes and practices of nurses related to maternal nutrition. It has the potential to lead to the betterment of nursing care, which in turn results in improved pregnancy outcomes in women.

## Introduction

Good nutrition is fundamental for good health and remains a priority in global public health agendas, following the adoption of sustainable development goals and the United Nations’ proclamation of a decade of action on nutrition 2016–2025 (Haddad et al. 2014). The World Health Organization ([WHO] 2016a) argues that nutrition plays a vital role in preconception, pregnancy and the post-partum period, and has a major impact on the quality of health of the woman and pregnancy outcomes. The importance of nutrition in the context of health is also highlighted in the recent evidence on the effects of maternal nutrition health and existing recommendations (Stephenson et al. 2018; WHO 2018). However, despite its importance, women of reproductive age, pregnant women, and children remain among the two billion people lacking essential nutrients globally (Hanson et al. 2015).

Iron and folate deficiencies are the most common causes of anaemia in pregnant women across sub-Saharan Africa, leading to preterm labour, low birth weight (LBW) and infant mortality (Balarajan et al. 2012; Procter & Campbell, 2014). In Lesotho, only 77.8% of women receive iron and folic acid supplements during pregnacy despite the rate of 35.5% prevalence of anaemia in pregnant women (Ministry of Health Lesotho and ICF International 2016; WHO 2017). Saronga et al. (2020) confirm that various micronutrient deficiencies during pregnancy have a link with increased risk of maternal morbidity and mortality. Furthermore, a healthy diet during pregnancy, which is characterised by adequate intakes of essential nutrients, reduces the risk of pregnancy complications (Saronga et al. 2020; Tryggvadottir et al. 2016).

Even though there have been many initiatives undertaken globally to address the issue of poor maternal nutrition, the majority are not tackling the problem at grassroots level, namely conception phase, but focus on nutrition during and after pregnancy (Ramakrishnan et al. 2012; WHO 2016b). This is no different to the results of a study conducted in Iceland on the association between healthy maternal dietary pattern and risk of gestational diabetes mellitus (GDM). The results indicated that dietary interventions in early pregnancy could be more effective if the basis for the choice of intervention was on pre-pregnancy nutrition status, and it was concluded that diet adherence in pregnancy was associated with lower risk of GDM, especially in at-risk women (Tryggvadottir et al. 2016).

According to the 2012 State of the World’s Mothers report, Save the Children (2012), maternal nutrition is important in the period from conception to the first 1000 days of life until a child’s second birthday. Literature further confirms that laying the foundation early and in the pre-pregnancy period is important for healthy growth, optimal child mental development, and it has benefits that would last for a lifetime (Christian et al. 2015; Cunha, Leite & Almeida 2015; Haddad 2013; Save the Children 2012). During this stage, epigenetic and early nutritional programming occur, as well as the foundations for a child’s future health in terms of growth and development (Bee et al. 2015; Geraghty et al. 2015; Save the Children 2012). The report continues to highlight that investing in nutrition develops a collective legacy for a sustainable world in 2030 by addressing the persistent problem of poor nutrition (Save the Children 2012).

Nurses, who play a vital role in promoting maternal nutrition are considered the backbone of maternity services. Hence, it is crucial for them to have adequate knowledge regarding the promotion of maternal nutrition to be able to provide effective nutritional services to women during pregnancy (Saronga et al. 2020). Therefore, this study aimed to assess the knowledge, attitudes and practices of nurses regarding nutrition in pregnant women.

## Problem statement

An optimal maternal nutrition status is crucial as the nutritional status of the infant is greatly dependent on the mother’s nutritional status before conception and during pregnancy (WHO 2016a). Over the years, there has been much evidence on the link between some nutritional deficits and increased maternal mortality (Manfredini 2020; Rukuni et al. 2016; Young et al. 2019). Yet, the magnitude of maternal mortality still remains a concern globally (Storeng & Béhague 2017). About 275 000 deaths are attributed to women who die from pregnancy or childbirth related complications yearly from preventable causes, including nutritional status-related complications (Diana, Wahyuni & Prasetyo 2020; Zureick-Brown et al. 2013).

Around 99% of these deaths, occur in developing countries (WHO 2013). According to the Ministry of Health Lesotho and ICF International (2016), the Lesotho Demography and Health Survey (LDHS) estimated maternal mortality in Lesotho to be 1024 per 100 000 live births, and in 2009 (2009), more than 40% of maternal mortality occurred in the district of Maseru. Malnutrition is a serious health problem affecting women during pregnancy and childbirth, which puts their children at a greater risk of poor physical and mental developmental path (Arrish, Yeatman & Williamson, 2016; Arrish, Yeatman & Williamson, 2017; Daba et al. 2013; Saronga et al. 2020). Although literature has documented the influence of nutrition on maternal health and pregnancy outcomes, there has been no study conducted to investigate the knowledge, attitude, and practices of nurses regarding maternal nutrition in pregnant women.

## Aim of the study

The study aimed to describe the knowledge, attitudes, and practices of nurses regarding maternal nutrition in pregnant women at a selected large hospital and filter clinics in Lesotho.

## Objectives of the study

- To assess the nurses’ knowledge regarding nutrition in pregnant women.
- To describe the nurses’ attitudes and practices regarding maternal nutrition.
- To determine the relationships between knowledge, attitudes, and practices of nurses regarding nutrition in pregnant women.

## Research design and method

### Study design

This was a descriptive cross-sectional study.

### Study setting

The study took place in a large hospital and its filter clinics in Lesotho. The hospital is in Maseru, the capital of Lesotho, which is the most populated district. This is a referral hospital for the whole country. It has a bed capacity of 425 and serves 453 606 thousand people (Bureau of Statistics Lesotho 2014). It has several reproductive health departments including obstetrics, gynaecology, outpatient clinics, an accident and emergency department and three refurbished and re-equipped primary healthcare clinics, which were the focus of the study.

### Study population and sampling

The study sample was selected using convenience sampling technique. The study included only nurses working in the selected departments, who met the inclusion criteria and who agreed to participate. The nurses who were not available during data collection and those who refused to participate were excluded. Sample size determination used a published table by Israel (2003). This was further confirmed by an online sample size calculator (Raosoft I 2004) using a precision of 5% and a 95% confidence level. A sample size of 122 was adequate to yield valid results.

### Research instrument

Data collection was carried out using a self-administered questionnaire. The researcher adapted the questionnaire developed by Sitati (2014), which assessed maternal nutrition knowledge, attitudes and practices among nurses working at Kenyatta National Hospital, Kenya. There were minor adjustments made to the questionnaire to achieve the objectives of the current study, in terms of key variables and number of questions. Following this, the questionnaire was pretested, and Cronbach’s alpha done to ensure reliability. The questionnaire comprised 14 questions on demographic data of respondents, 15 multiple-choice questions on the nurses’ knowledge regarding nutrition in pregnant women, 10 questions on attitudes and 15 on nurses’ practices regarding nutrition in pregnant women. For knowledge regarding nutrition, respondents were required to choose the correct answer; a score of 1–7 was considered poor knowledge, while 8–15 was considered good knowledge. The attitude scale consisted of 10 items on a four-point Likert scale ranging from ‘strongly agree’ as score 4, to ‘strongly disagree’ as score 1. Scoring for the items involved summing the responses to a minimum score of 10 and maximum score of 40. Fifteen questions assessed the nurses’ practices on a four-point scale, ranging from 0 (never) to 3 (always), with ‘never’ regarded as poor practices while ‘always’ denoted good practices.

### Validity and reliability

This study used content and face validity. This was with peer-reviewed literature and by relating objectives of the study to the specific questions on the instrument. A pre-test of the questionnaire was carried out on 5% of the sample size in a similar setting to assess its reliability. Cronbach’s alpha for the sections of the questionnaire were as follows: knowledge, 0.25, attitude, 0.86 and practice 0.81 while the acceptable value was set at 0.05.

### Data collection procedure

Data collection took place over a period of four weeks in November–December 2017. After obtaining ethical clearance and permission from selected institutions, appointments were arranged with managers to contact the respondents. An information sheet describing the details of the study was given to the respondents. The information was also explained verbally. After obtaining written informed consent, the researcher distributed the questionnaires to the nurses and gave guidance on how to complete them. It took about 10 min to complete the questionnaire, and the respondents could complete it at their own convenience.

### Data analysis

Data were entered and subsequently analysed using the Statistical Package for Social Sciences (SPSS), version 25. Descriptive statistics in the form of percentages, item scores, means, mode, medians, and standard deviations (SDs) have been used to communicate the data using frequency tables. Non-parametric tests and Spearman’s rho tests determined the association between variables, and the Kruskal Wallis and Mann Whitney U tests were conducted to find out if there were any differences between variables. Fisher’s Exact and Chi-square tests determined the significance in the relationships between variables.

### Ethical considerations

The granting of ethical approval by the University of KwaZulu-Natal Research Ethics Committee and the Lesotho Ministry of Health Research Coordination Unit was according to the Declaration of Helsinki (Reference number: HSS/1838/017M). After obtaining ethical clearance, the researcher sought permission from the director of the selected hospital to conduct the study. The study had no physical, social, or psychological risks. The respondents received all the information about the study verbally and in writing. The participation in the study was voluntary and there was no coercion used. There was no identifying information used on the questionnaires. Hard copies of raw data in the form of completed questionnaires were scanned to the researcher’s computer and papers were destroyed by shredding. The soft copies of data analysis spreadsheets, other related documents and the scanned copies remained in a password protected folder on the researcher’s computer, where it will be stored for a period of five years, according to the policy of the university of study.

## Results

### Demographic characteristics of the respondents

The respondents’ demographic variables included age, gender, marital status, nursing category, academic qualification, work experience, department, nursing experience in the department, and maternal nutrition training. The demographic profile of the study population is shown in Table 1.

**TABLE 1:** Sample characteristics (*n* = 120).

Variables	*N* = 120	%
**Age (*N* = 120)**		
20–29	62	51.6
30–39	35	29.0
40–49	14	11.6
50–59	9	7.5
**Gender (*N* = 120)**		
Female	104	86.7
Male	16	13.3
**Marital status (*N* = 120)**		
Married	78	65.0
Unmarried	35	35.0
**Academic qualifications (*N* = 120)**		
Certificate	29	24.2
College diploma	71	59.2
University degree	15	12.5
University diploma	5	4.1
**Category (*N* = 120)**		
Registered nurses	90	75.0
Nursing assistants	30	25.0
**Work setting (*N* = 120)**		
Filter clinic	49	40.8
Hospital wards	47	39.2
Outpatient and accidents and emergency	24	20.0
**Department (*N* = 120)**		
Maternity	46	38.3
Gynaecology	14	12.5
Accidents and emergency	23	18.3
Primary health care	37	30.8
**Nursing experience in years (*N* = 120)**		
˂ 1	30	25.0
1–2	24	20.0
3–4	19	15.8
˃ 5	47	39.2
**Short course maternal nutrition training (*N* = 120)**		
Trained	107	89.2
Not trained	13	10.8
**Length of short maternal nutrition course attended (*N* = 120)**		
Never attended	17	14.2
5 days	62	51.7
6–10 days	36	30.0
15 days	5	4.1
**Regularity of the maternal nutrition training (*N* = 120)**		
Quarterly	22	18.3
Bi-annually	3	2.5
Annually	17	14.3
Once every three years	8	6.7
Never	2	1.7
Don’t know	68	56.7
**Last maternal nutrition training attended (*N* = 120)**		
Never attended	35	29.2
1–2 years	68	56.7
3–4 years	10	8.4
˃ 5 years	7	5.7

### Knowledge regarding maternal nutrition

Overall, the results showed that the nurses had good knowledge about maternal nutrition with a mean (M) knowledge score of 10.14 out of a possible 15, with an SD of 1.9. The results show that most of the questions that assessed the knowledge regarding maternal nutrition were answered correctly. For instance, out of 120 nurses, 99.2% (*n* = 119) gave a correct answer to the statement, ‘To avoid constipation, the pregnant woman should increase her intake of whole-grain, vegetables and fruits’. Only four questions regarding maternal nutrition knowledge were answered incorrectly. The nurses showed poor knowledge on the recommended time for storage of iron and calcium 23.3% (*n* = 28), recommended daily energy intake during the last two trimesters of pregnancy 45% (*n* = 54), optional weight gain during pregnancy 24.2% (*n* = 29) and cause of physiological anaemia during pregnancy 17.5% (*n* = 21). Table 2 displays the results.

**TABLE 2:** Nurses’ responses on maternal nutrition knowledge.

Item	Correct		Incorrect	
	*n*	%	*n*	%
1. A maternal practice that can be harmful to the foetus is smoking.	118	98.3	2	1.6
2. To produce a healthy infant, a mother should ideally have an adequate diet.	92	76.7	28	23.3
3. The third trimester is the main time for storage of iron, fat, and calcium.	28	23.3	92	76.7
4. Infants born after normal gestation length but weighing less than 2.5 kg are labelled small for gestational age.	73	60.8	47	39.1
5. A pregnant woman needs to increase her energy intake by about 300 Kcals/day during the last two trimesters of pregnancy.	54	45.0	66	55.0
6. An energy source to avoid in pregnancy and lactation is alcohol.	106	88.3	14	11.7
7. An increased requirement for folate and vitamin B-12 during pregnancy is related to their roles in the synthesis of red blood cells.	79	65.8	41	34.1
8. Weight gain in pregnancy for healthy women should usually be at least 12 to 15 kg.	29	24.2	91	75.8
9. The practice of eating dirt or laundry starch during pregnancy is called pica.	108	90.0	12	10.0
10. Maternal age of 30–35 years is not likely to pose a risk to maternal and foetal health during pregnancy and childbirth.	88	73.3	32	26.7
11. The risk of delivering a premature or small for gestational age infant increases with maternal smoking, alcohol consumption, and illegal or improper drug use.	111	92.5	9	7.5
12. The desirable goal of a successful pregnancy is a gestational period longer than 37 weeks and birth weight greater than 2500 g.	100	83.3	20	23.8
13. To avoid constipation, the pregnant woman should increase her intake of whole-grain cereals, vegetables, and fruits.	119	99.2	1	8.0
14. Physiological anaemia of pregnancy results from an increase in the mother’s blood volume.	21	17.5	99	89.6
15. Iron and folic acid supplements (Rifas-Shiman et al. 2009) should be taken by pregnant women from conception to delivery.	101	84.1	19	15.9

### Overall knowledge score

The knowledge scale had 15 items. The possible minimum score was zero and maximum score was 15. The minimum score for the respondents was five and the maximum score was 14. The mean knowledge score was 10.14 with an SD of 1.9. Hence, it can be concluded that the respondents had good knowledge about maternal nutrition.

### Association between knowledge and demographic characteristics of nurses

There was a significant association of the level of knowledge found with the nursing category, with *p*-value 0.003 and academic qualification at *p*-value 0.009 (Table 3). Performing a Chi-square test between the knowledge regarding maternal nutrition and the nursing categories showed values = 8.733, df 1, and *p* = 0.003. This meant the higher the nursing category the better the knowledge, and lower the nursing category, the poorer the knowledge. To test the association between the respondents’ knowledge regarding maternal nutrition and the academic qualifications, a Fisher’s Exact test showed the value = 10.344, df 0, and *p* = 0.009. Accordingly, this showed that the higher the academic qualification the better the knowledge regarding maternal nutrition. Therefore, there was a significant association between the knowledge regarding maternal nutrition and nursing category and academic qualification.

**TABLE 3a:** Association between knowledge and demographic variables.

Category of nurses	Knowledge level						*p*
	Poor knowledge		Good knowledge		Total		
	*n*	%	*n*	%	*n*	%	
Nursing assistants	8	26.7	22	73.3	30	100	0.003
Registered nurses	6	6.7	84	93.3	90	100	

**Total**	**14**	**11.7**	**106**	**88.3**	**120**	**100**	

**TABLE 3b:** Association between knowledge and demographic variables.

Qualification	Knowledge level						*p*
	Poor knowledge		Good knowledge		Total		
	*n*	%	*n*	%	*n*	%	
Certificate	7	24.1	22	75.9	29	100.0	0.009
College diploma	5	7.0	66	93.0	71	100.0	
University diploma	2	40.0	3	60.0	5	100.0	
Bachelor’s degree	0	0.0	15	100.0	15	100.0	

**Total**	**14**	**11.7**	**62**	**88.3**	**120**	**100.0**	

### Attitude of nurses towards maternal nutrition in pregnant women

Ten items on a four-point Likert scale, which ranged from ‘strongly agree’ to ‘strongly disagree’, measured the nurses’ attitudes towards maternal nutrition. Because of low response rates for certain questions, and to make a meaningful interpretation, the scale was reduced to only two options, with the options of ‘strongly agree’ and ‘agree’ combined, and ‘strongly disagree’ and ‘disagree’ being combined. The ‘agree’ responses were regarded as positive attitude, and ‘disagree’ responses as negative attitude towards maternal nutrition. Table 4 presents the results on attitudes.

**TABLE 4:** Responses to questions on attitude of nurses towards maternal nutrition.

Item on attitude of nurses regarding maternal nutrition	Agree		Disagree	
	*N*	%	*N*	%
1. It is important to screen for maternal nutrition risk factors of all women admitted to the ward.	118	98.3	2	1.7
2. Screening for maternal nutrition risk factors in pregnant women at the clinic and wards is one of the responsibilities of the nurse.	118	98.3	2	1.7
3. Underweight and overweight women experience more complications during pregnancy and delivery than normal women.	109	90.8	11	9.2
4. All pregnant women should be knowledgeable about the need for an adequate and nutritional diet.	117	97.5	3	2.5
5. All women should be encouraged to take iron and folic acid supplementation (Rifas-Shiman et al. 2009) during pregnancy irrespective of their haemoglobin level.	116	96.7	4	3.3
6. All women should be counselled on adequate and healthy weight gain during pregnancy.	116	96.7	4	3.3
7. All women at risk, including adolescents with HIV, in emergency situations, should receive nutritional support.	114	95.0	6	5.0
8. Pre-pregnancy nutrition influences a woman’s ability to conceive. It determines foetal growth and development as well as the health of the woman.	109	90.8	11	9.2
9. Good maternal nutrition is important for a successful pregnancy, child delivery, and lactation.	116	96.7	4	3.3
10. Educating women on the importance of healthy eating during pregnancy is one of the responsibilities of a nurse.	114	95.0	6	5.0

### Overall attitude score

The attitudes scale had 10 items. The possible minimum score was 10 and the maximum was 40. The mean attitude score was 36.98 with a SD of 3.98. Thus, on average, the results indicate that the respondents showed a positive attitude towards maternal nutrition. Out of 120 nurses, only 0.8% (*n* = 1) had a score of less than 25, which indicated negative attitude, while the rest (99.2%: *n* = 119) fell between 26 and 40, positive attitudes. Summarising the results of the 10 questions on attitudes of nurses regarding nutrition in pregnant women, most of the respondents (98.3%: *n* = 118) agreed it was not only important to screen pregnant women for nutritional risk factors, but it was the nurses’ responsibility to do so. It was also good to note that 95% (*n* = 114) of the nurses agreed that educating woman on the importance of healthy eating during pregnancy was one of the responsibilities of a nurse.

### Practices of nurses regarding maternal nutrition

To assess the nurses’ practices regarding maternal nutrition, 15 questions asked about the activities performed with regard to the promotion of such, and how often nurses performed certain activities when screening for maternal nutrition risk factors. The responses were based on a four-point Likert scale, with options ranging from ‘never’ to ‘always’. Because of low response to some categories, the categories of ‘never’ and ‘rarely’ were combined under the response ‘rarely’, while ‘sometimes’ and ‘always’ were combined as ‘always’. The results showed that out of the 120 respondents, 77.5% (*n* = 93) reported always using nutritional guidelines when discussing a nutritional plan for pregnant women. The nurses also agreed to always taking vital signs and patient’s diet history to screen for nutritional risk factors in pregnant women, 93.4% (*n* = 112) and 90.0% (*n* = 108), respectively. One hundred per cent (*n* = 120) of nurses agreed that they always encouraged pregnant women to take iron supplements and folate daily. The nurses also agreed to consult a dietitian (68.4%: *n* = 82) and discuss diet options with the patient (95.9%; *n* = 115) in cases where pregnant women were not taking adequate diet. Lastly, 88.3% (*n* = 106) agreed that they always weighed patients for obvious weight loss, poor appetite, and reduced food intake (Table 5).

**TABLE 5:** Nurses’ responses to questions on maternal nutrition practices.

Item	Always		Rarely	
	*n*	%	*n*	%
I use nutritional guidelines when discussing nutrition plan for pregnant women	93	77.5	27	22.5
**Types of actions taken to screen for nutritional risk factors in pregnant women**				
I take vital signs like blood pressure, pulse rate, temperature	112	93.4	8	6.7
I assess if weight gain is within recommendations	108	90.0	12	10
I take mid upper arm circumference (MUAC)	96	80.0	24	20
I take the diet history	108	90.0	12	10
I take the blood biochemistry	84	70.0	36	30
**Type of information provided to the pregnant woman**				
I educate pregnant women in the ward/clinic on the importance of eating a healthy and well-balanced diet	117	97.5	3	2.5
I encourage pregnant mothers to take nutritional supplements containing Iron and Folic Acid (Rifas-Shiman et al. 2009) daily during their duration of pregnancy	120	100	0	0
I discuss the nutritional status and nutritional management of pregnant mothers with other team colleagues during ward rounds or clinic visits	104	86.7	16	13.3
**Actions taken when the patient is not taking adequate diet**				
I consult the dietitian	82	68.4	38	31.7
I refer to the doctor	98	81.7	22	18.3
I discuss with the patient the possible diet options	115	95.9	5	4.2
**Reasons for assessing weight gain**				
I assess weight gain for medication purposes	97	80.8	23	19.2
I assess weight gain for patient’s medical condition	105	80.8	15	12.5
I weigh the patient for obvious weight loss, poor appetite, and reduced food intake	106	88.3	14	11.7

### Overall practice score

The responses ranged on an ordinal scale, from zero to three (0 = never, 1 = rarely, 2 = sometimes and 3 = always); the minimum possible score was zero and the maximum score was 60. A higher frequency score of an activity indicated good maternal nutrition practices and a lower frequency score indicated poor practices. The mean score of respondents was 36.33, the median was 37, and the mode 37. The SD was 6.7.

### Association between institution type, department, and maternal nutrition practices

A cross tabulation of the respondents’ institution type and practice showed that those from the filter clinics have good practices compared to those from hospital wards and Accident and Emergency Department (A&E); the majority (54.7%: *n* = 41), having good practice, and only 17.8% (*n* = 8) having poor nutrition practices. The Pearson Chi-square shows a significant association; Chi-square = 19.144, *df* = 2, and *p* = 0.000. There was a significant association found regarding practices with institution where practice took place, with *p*-value 0.000, and department at *p*-value 0.002. Pearson Chi-square shows a significant association; Pearson value = 14.926, *df* = 3, *p* = 0.002. Table 6 presents the results.

**TABLE 6a:** Type of institution, department, and maternal nutrition practices.

Type of institution	The maternal nutrition practices				*p*
	Poor		Good		
	*n*	%	*n*	%	
Hospital ward	28	62.2	19	25.3	0.000
Hospital out-patient/A&E	9	20	15	20	
Filter clinics	8	17.8	41	54.7	

**Total**	**45**	**37.5**	**75**	**62.5**	

**TABLE 6b:** Type of institution, department, and maternal nutrition practices.

Department	The maternal nutrition practices				*p*
	Poor		Good		
	*n*	%	*n*	%	
Maternity	24	53.3	22	29.3	0.002
Gynaecology	8	17.8	7	9.3	
Out- patient/A&E	8	17.8	14	18.7	
Primary health care	5	11.1	32	42.7	

**Total**	**45**	**37.5**	**75**	**62.5**	

### Interrelationships between knowledge, attitude, and practice

The Pearson correlation test was conducted to find out the associations between knowledge, attitude, and practice. The results showed a relationship between attitude and practice, with a correlation coefficient of *r* = 296, *n* = 120, and *p* = 0.001; other tests did not show the relationship (Table 6).

**TABLE 7:** Correlation between knowledge, attitude, and practice.

Pearson correlations	Knowledge	Attitude	Practices
**Knowledge**			
Pearson Correlation	1	0.112	−0.125
Sig. (2-tailed)	-	0.225	0.174
*N*	120	120	120
**Attitude**			
Pearson Correlation	0.112	1	0.296**
Sig. (2-tailed)	0.225	-	0.001
*N*	120	120	120
**Practices**			
Pearson Correlation	−0.125	0.296**	1
Sig. (2-tailed)	0.174	0.001	-
*N*	120	120	120

** , Correlation is significant at the 0.01 level (2-tailed).

## Discussion

It is noteworthy that the response rate for this study was very high, at 98.0%. The results showed that the majority of the respondents were younger than 40 years, which is similar to the previous studies (Demilew & Nigussie 2017; Stender et al., 2013). Just as females tend to dominate the nursing profession (Bonilla et al. 2016; Meadus & Twomey 2011; Sitati 2014; Stender et al. 2013; Wondimagegne & Shele 2015), the majority of the respondents in the present study were females.

In a study conducted in Northwest Ethiopia, Demilew and Nigussie (2017) assert that nurses working in healthcare centres should have adequate knowledge on the provision and prescribing practice of nutritional supplements before and during pregnancy. These authors argue that this is where most of the routine clinical, health education and screening of pregnant women takes place (Demilew & Nigussie 2017). Therefore, the findings of this study are encouraging as the first point of entry for pregnant women seeking maternal healthcare begins in the health centres where 40.8% (*n* = 34) were allocated. Symington et al. (2018) confirm that maternal nutrition interventions are mostly provided by nurses. Regarding the age of nurses younger than 40 years, Laschinger et al. (2016) encourage keeping the young generation of nurses as the current nursing workforce is aging.

The results of this study showed that the knowledge of nurses regarding maternal nutrition was good. Most of the respondents (89.2%) stated that they had received training in maternal nutrition. These results match with the findings of previous studies conducted in other countries including Ghana and Finland, which revealed that almost all the study participants had completed and passed the nutrition course required in their training (Demilew & Nigussie 2017; Ilmonen, Isolauri & Laitinen 2012; Mogre et al. 2017). Most of the current study’s respondents (75%) of registered nurses and 25% of nursing assistants, had covered the topic of maternal nutrition during their nursing training. More than three quarters had attended a short in-service course, which is consistent with previous studies’ findings (Mogre et al. 2017; Ilmonen et al. 2012). The majority (88.3%) had good knowledge of maternal nutrition, which could be because of the short courses. This is in contrast with the results from other studies, where most nurses were inadequately prepared to screen for maternal nutritional risk factors because of lack of knowledge (Arrish, Yeatman & Williamson 2014; Arrish et al. 2016; Nurdan 2013; Wondimagegne & Shele 2015).

Various studies have demonstrated that in general nurses have positive attitudes towards maternal nutrition (Arrish et al. 2016, 2017; Bjerrum, Tewes & Pedersen 2012; Kim & Choue 2009; Mogre et al. 2017). Similarly, the results of the present study showed that most of the respondents (99.2%; *n* = 119) had positive attitudes towards the factors that could improve maternal nutritional status. For example, the respondents agreed that good maternal nutrition is important for a successful pregnancy, child delivery and lactation (96.7%: *n* = 116). This is in support of what Symington et al. (2018) state, that adequate nutrition during pregnancy is important to ensure optimal birth outcomes, maternal health and offspring development.

This study’s results corroborate previous findings that nutrition-related practices must remain adequate for improved health and uncomplicated deliveries with the use of maternal anthropometric tools (Sunsaneevithayakul et al., 2014). In addition, there was a significant relationship between institution type and department on practice, with nurses from the filter clinics and primary healthcare showing good practices than those that were hospital-based, *p* = 0.000 and 0.002, respectively. This finding is consistent with the findings of Arrish et al. (2014), which showed that nurses have a great opportunity to screen risks and provide nutritional information as they are the first contact that pregnant women have during antenatal visits. However, other studies found that knowledge and attitudes did not align with practice (Arrish et al., 2016; Yalcin et al. 2014).

In addition, most respondents (77.5%: *n* = 93) reported that their maternal nutrition practices were guided by nutritional guidelines. This is in contrast with what Wilkinson, Poad and Stapleton (2013) found in their study conducted in Queensland, that maternity health professionals’ lack of guideline knowledge influenced their consultation with pregnant women. Conversely, in a study conducted in Liverpool (England, UK) and Ulster (Northern Ireland, UK), McCann et al. (2018) reported the following: ‘There is a mismatch between what the evidence‐base tells us, what directive policy and clinical guidelines provide, and what midwives actually do in practice’. Consequently, a need to implement literature to understand the barriers to optimal healthcare delivery and inform clinical practice using research evidence was highlighted (McCann et al. 2018).

Practices to promote nutrition during pregnancy are among the key actions recommended by the South African Government to promote optimal early childhood development and to eliminate poverty (Symington et al. 2018). It was noteworthy that in the current study, available maternal nutrition guidelines guided the nutritional practices in pregnant women. Another nutrition practice related to the finding from this study was that 80% (*n* = 96) of nurses agreed they take mid upper arm circumference (MUAC) when they screen pregnant women for nutritional risk factors; this is consistent with the international standards for anthropometric measurement (Marfell-Jones, Stewart & De Ridder 2012).

Furthermore, the maternal nutrition practices of nurses were good in this study, with a mean score of 36.33 out of a possible 60; results also showed that they had good knowledge, mean score of 36.98 out of a possible 40 and positive attitude with 36.98 out of possible 40. This finding supports Saronga et al. (2020), that nutrition services normally provided to pregnant women by nurses include nutrition education, provision of iron and folate supplements, weight measurements, dietary assessment and monitoring of haemoglobin levels. The results also showed that the nurses’ practices were good, and they indicated that nutritional guidelines guided them. This finding was in contrast with what McCann et al. (2018) found in their study, that there was a mismatch between what the clinical guidelines recommended, and what was practised. However, Wilkinson et al. (2013) argued that nurses’ may have lacked guideline knowledge.

When assessing the relationship between the study variables, the results showed no relationship between knowledge and the other variables, but there was a relationship between attitudes and practices of nurses with a correlation coefficient of *r* = 296, *n* = 120, and *p* = 0.001. Out of 120 nurses, 99.2% (*n* = 119) had positive attitude towards maternal nutrition, with a score range of 26–40 out of a possible 40 while the and 62.5% (*n* = 75) scored 36–60 out of a possible 60. This means that the maternal nutrition practices of nurses were influenced by their positive attitudes and not by their knowledge. The results are similar to what Tomar et al. (2021) found in their study on knowledge, attitude and practices regarding COVI-19, that practice can be influenced by varied factors, including acceptance of an intervention. It was noteworthy that 98.3% (*n* = 118) of nurses in the current study agreed that it was important to screen pregnant women for nutrition risk factors and that it was their responsibility to do so.

## Limitations

The data collection used self-completed questionnaires, which allowed the respondents to complete them in their own space and time, which could have reduced reliability of the results. Despite the obvious advantages of using self-completed questionnaires, forgetfulness, overly positive assessment of one’s abilities and social desirability bias might decrease the reliability of the results (Lajunen & Summala 2003; Walentynowicz, Schneider & Stone, 2018). The study took place in only one institution, and therefore the results need interpreting with caution and not generalised to the entire nurse population in maternal health in Lesotho. The results may, however, contribute to the body of literature related to maternal nutrition as the nutrition surveillance system for maternal and child health maternal and child health in Lesotho is inconsistent despite the country’s high rate of maternal and child mortality ratios (Mugomeri 2018).

## Recommendations

Hospital policies should incorporate nutritional practice guidelines that delineate the nurses’ role in nutritional risk screening, education and/or intervention, particularly in the hospital wards and maternity department. Nurses must refer patients to dietitians if screening shows a nutritional risk. There is a need for more research on maternal nutrition guidelines implementation in all healthcare institutions or settings to standardise practices. Standardised nutritional practices may influence the clinical outcomes of pregnant women admitted in hospital wards and primary healthcare settings.

## Conclusion

This study showed that the knowledge of nurses regarding maternal nutrition was good. Their attitudes were positive, and their practices were good but varied among departments. This denotes that adequate knowledge and positive attitude can translate into good practices. Therefore, there is a need for strategies to enhance knowledge translation to improve the quality of maternal nutritional care services offered by nurses.

### Primary contribution of the study

This study highlights the role that nurses play in the nutritional care of pregnant women. The results may contribute to the body of literature related to the knowledge, attitude and practice of nurses with regard to maternal nutrition evaluation, and has the potential to lead to the betterment of nursing care, which in turn results in improved pregnancy outcomes in women.
